# The Reason Beer Makes You Pee and Why You Should Abstain before Orthopedic Surgery

**DOI:** 10.3390/nu15071687

**Published:** 2023-03-30

**Authors:** Matteo Briguglio

**Affiliations:** Laboratory of Nutritional Sciences, IRCCS Orthopedic Institute Galeazzi, 20161 Milan, Italy; matteo.briguglio@grupposandonato.it

**Keywords:** fermented foods, alcoholic beverages, brewing, physiological phenomena, diuresis, sodium, potassium, calcium, orthopedic surgery, hip replacement arthroplasty

## Abstract

Hydration practices in the view of hip, knee, or spine surgery instruct patients to avoid caffeinated drinks, alcoholic beverages, and sugar-sweetened drinks because they adversely impact body fluid homeostasis. However, some patients might be inclined to not include beer among the prohibited beverages because of its low alcohol content and conflicting evidence about its rehydrating effects. The author of this opinion article discusses the shreds of evidence that establish beer as a drink to avoid prior to orthopedic surgery.

## 1. Preoperative Preparation for Patients Undergoing Orthopedic Surgery

Prior to hip, knee, or spine surgery, patients meet with an orthopedic surgeon, an anesthetist, and a nurse for a health assessment, a disease-related risk calculation, a scheduling of the intervention, and a planning of prehabilitation protocols. From the patients’ perspective, this period between the orthopedic consultation and the day of surgery should not be a passive waiting but ought to be an opportunity to follow instructions and adopt habits that enhance recovery after surgery [1]. Alcohol abstention is an aspect of particular importance, upon which the maintenance of blood pressure and fluid homeostasis depends [2]. Alcoholic beverages, along with caffeinated and sugar-sweetened drinks, negatively impact the physiology of water balance and, therefore, should be avoided. A large proportion of seniors regularly consume a moderate amount of alcohol, and the aging process is known to increase the risk of dehydration [3,4]. Therefore, old patients, in particular, ought to be advised to make choices on what to drink in view of the upcoming operation, including refraining from dehydrating drinks and, thus, preferring water as early as feasible, given that the restoration of fluids and electrolyte losses might take a few days [5]. Nevertheless, there is one beverage that presents controversy: beer. Some reports suggest that beer does not seem to cause dehydration [6] but instead aids in hydration [7], and that it shall not be renounced after exercise [8]. It is the author’s idea that it is mandatory to clarify that the indication of abstention from dehydrating beverages before orthopedic surgery also includes beer. In this opinion article, the author briefly introduces the brewing methods of beer, debates the reasons why beer appears to be a diuretic drink, and contextualizes the argument to support beer as a drink to avoid prior to orthopedic surgery.

## 2. What Is Beer?

Beer is crafted from the skillful processing of four basic ingredients, including a source of starch, a fermenting agent, a flavoring agent, and a solvent. The raw materials chosen to fill these roles have been mostly germinated barley, yeast (*Saccharomyces cerevisiae* for ales or *Saccharomyces pastorianus* for lagers), the edible flowers of *Humulus lupulus* L., and water. Recipe adaptations encompass the selection among different varieties of barley (e.g., the high-starch, low-protein, two-row *Hordeum distichon* L. or the high-protein, low-starch, six-row *Hordeum vulgare* var. *hexastichum*) or an assortment of roots and tubers, such as cassava or yams [9,10]. The formula used largely depends on local food security and cultural roots. For instance, rice is used in Asian countries, sorghum or millet are preferred in some regions of Africa, parsnip is used in Britain and Ireland, and wheat is now being used worldwide. Buckwheat or oat can also be used, mainly for gluten-free beers. There are also considerable variations in the flavoring agents used, which include dandelion in England, woodruff in Berlin, dates in Nigeria, and ginger in the Greek island of Corfu and in the Caribbean Island nation of Jamaica [11,12]. These ingredients provide different varieties of beers their aromas and colors, and technological enhancements, such as foam stabilization, are also employed [13]. Given the complexity of ingredients, brewing methods, and final products [14,15], it is challenging but also superfluous to debate all sorts of beers. In the context of this opinion article, the emphasis will be on the traditional recipe that gives life to a beer with a moderate alcohol content and no flavoring agents other than hops (Table 1). The non-alcoholic fraction of beer is of particular interest because it is often regarded as a source of bioactive compounds, which may have a role in the alleged alteration of hydration status.

## 3. Why Beer Makes You Pee

Drinking beer causes the drinker to “break the seal”. The volume of urine is primarily determined by the volume of ingested water, which corresponds to around 94% of beer volume. A pint (0.568 L) of beer provides almost equal to three glasses of water, which is a sufficient amount of fluid to initiate urine production. Another element to consider is the energy supplied by beer, which is around 200 kcal per pint, thus generating an additional 24.6 mL of metabolic water [5]. The average content of ethyl alcohol in a pint is 23 g. This compound is notorious for decreasing hypothalamic vasopressin mRNA expression and the content of this hormone in the posterior pituitary; reducing the stimulus of related V2 receptors in the basolateral membranes of the collecting tubules and ducts; decreasing the long-term gene expression of local aquaporins; halting the short-term translocation of these proteins from the intracellular vesicles to the apical membranes; and thus reducing water permeability [16,17]. The extent of the triggering of water diuresis by alcohol depends on alcohol tolerance; however, whilst not substantially altering long-term water volume in serum [18], ethanol independently affects osmolality [19] and further elicits short-term urine production [20]. This organic compound is anecdotally listed among bladder irritants, probably due to sensory disorders of the urinary tract, together with the thinning and weakening of the bladder wall, seen in heavy drinkers [21]. The electrolytic fraction of beer is another factor influencing the body fluid status [22]. Sodium has the function of retaining body fluids and restoring lost plasma volume, but the quantities of this alkali metal in beer are ten times smaller than potassium, which is contrariwise considered a diuretic agent that decreases extracellular fluid volume, angiotensin activity of vascular smooth muscle cell receptors, and peripheral resistance [23]. An essential mineral ratio in favor of urine production is, therefore, generated [24,25]. It also seems that the more a beer is acidic and bitter, with an astringent taste, or in general capable of provoking intense sensory stimuli, the more it is considered thirst quenching [26,27]. Consequently, drinking a regular beer perhaps satisfies thirst a little and leads to more time being spent drinking, time that is per se marked by a physiological hourly urine output of 1 mL per kilo of body weight in addition to the amount determined by the ingested water. The time between the first sip and the breaking of the seal also depends on the stomach’s fullness. Delayed gastric emptying due to disgust with the drink [28] or concomitant ingestion of salty or energy-dense food [29] is associated with reduced urine output. As a result, sipping a regular, refreshing, moderately alcoholic, and mildly flavored beer on an empty stomach could be the perfect formula to break the seal early. Lastly, barley and hops are sources of beer phytochemicals, some of which, such as phenolic acids and flavonoids, boast diuretic effects [30,31,32,33]. In the case of beers with plant alkaloids, such as methylxanthines in mate beers [34], there is a further increase in diuresis and natriuresis [5].

## 4. Beer Abstention before Orthopedic Surgery

During major orthopedic surgery, important changes in patients’ hydration status occur, and, hence, patients must have a good hydration status once positioned on the operating table. The abovementioned evidence suggests that ethanol and non-alcoholic constituents of beer may adversely impact body fluid homeostasis, creating a good sense for why patients undergoing orthopedic surgery must withdraw from drinking beer. Alcohol has long been the best-known reason for beer abstention because it interferes with the metabolism of many drugs used for chronic illnesses, unfavorably affects the blood-clotting system, and is a source of extra calories that could influence healthy eating habits and weaken nutritional status [35,36]. Furthermore, numerous beer phytochemicals interact with drugs used for anesthesia [37,38,39]. This is not to distrust the consumption of beer per se. Yet, it is important during the preadmission visit to highlight that beer and possible alcohol-free alternatives ought not to be counted among the permitted drinks, especially in the case of frail individuals with cardiovascular or renal ailments that make the body system more prone to decompensation [40,41]. There are no clinical studies involving orthopedic populations that can support the author’s recommendation. The experiments that have investigated the effects of beer drinking on hydration have mostly focused on the sports field [42]. There is a substantial lack of evidence from randomized controlled trials on how beer might truly affect first urinary latency, urinary electrolyte excretion, urinary pH and osmolality, or serum levels of sodium and potassium. Nevertheless, two things are important to remember. First, diuresis depends on the state of hydration of the drinker who, if already hypohydrated before starting drinking, would tend to produce less urine [16]. Second, drinking excessive quantities of beer alone leads to a unique syndrome of low sodium level in the blood called “beer drinkers’ hyponatremia” or “beer potomania” [43].

## 5. Final Considerations

In conclusion, beer is a fascinating beverage that master brewers create from common ingredients that are subjected to a succession of the delicate steps of malting (steeping, germinating, and kilning of barley), milling, steeping with water and mashing (brewing), lautering, boiling with hops, clarifying and cooling, fermentation, maturation of green beer, filtration, stabilization, and packaging, thus giving birth to a noble and versatile drink that acts as a social lubricant [44]. The yearly consumption of beer in Italy reaches 30 L per capita, but in some central European countries, the quantity exceeds that amount by three times. A moderate consumption of beer seems not to harm musculoskeletal health [45], but further studies will elucidate the concerns regarding the association between its phosphorus content [46], its high glycemic index of almost 90 [47,48], and bone resorption. Conversely, other constituents seem to exert positive health effects, such as melanoidins in dark beers, which limit the production of secondary lipoxidation products during digestion, and 8-prenylnaringenin, which behaves like a weak 17β-estradiol [49]. Notably, it is possible that some phenomena derive from a synergy of the non-alcoholic fraction of beer with ethanol, which disappear in alcohol-free beers [50]. Despite these benefits, the maintenance of fluid homeostasis is one of the most important issues of perioperative medicine, and it is better to be safe than sorry. It is the author’s prudent opinion that all patients undergoing orthopedic surgery, regardless of sexual orientation and age, should completely abstain from consuming beer at least two weeks before the operation. It is not enough to restrict its consumption because the health effects might not be proportional to the quantity drunk, as in the case of the risk–severity paradox that occurs in females compared to males [51]. The advice to withhold should also cover alcohol-free alternatives because, on par with herbal remedies [52,53], it is reasonable to believe that the preventive abstention outweighs the potential consequences on diuresis, electrolytes, blood pressure, and sedation. The authors’ opinion could definitely be transferred to other surgical specialties as well since the maintenance of fluid homeostasis is a prerogative in every major surgical procedure.

## Figures and Tables

**Table 1:** Constituents of a regular beer.

Constituent	Content (100 mL)
**Energy**	
Calories (kcal)	27.0–43.0
**Water**	
H_2_O (g)	91.96–95.9
**Alcohol**	
Ethanol (g)	3.8–4.6
**Carbohydrates**	
Sugars (g)	2.2–8.6
**Proteins**	
Proteins (g)	0.2–0.5
**Minerals**	
Sodium (mg)	1.2–4.0
Potassium (mg)	27.0–55.0
Chloride (mg)	17.0–20.0
Magnesium (mg)	4.0–9.6
Calcium (mg)	3.5–7.0
Phosphorus (mg)	13.0–19.0
**Bioactive compounds**	
Polyphenols (mg)	3.60–48.90
Phenolic acids (mg) ^1^	0.23–7.42
Flavonoids (mg) ^2^	0.1–2.28
Lignans (mg) ^3^	tr–0.05

Quantitative ranges were collected from multiple online sources consulted on 21 February 2023: the food composition database for epidemiological studies in Italy (bda-ieo.it), the Swiss food composition database (naehrwertdaten.ch), the food composition database of the European Food Safety Authority (efsa.europa.eu/en/data-report/food-composition-data), the FoodData Central of the United States Department of Agriculture (fdc.nal.usda.gov), the database on polyphenol content in foods (phenol-explorer.eu), and the Canadian FooDB (foodb.ca). ^1^ Phenolic acids comprise hydroxybenzoic acids (gallic, gallic 3-O-gallate, syringic, and vanillic acids), hydroxycinnamic acids (caffeic, ferulic, *p*-coumaric, and sinapic acids), and hydroxyphenylacetic acids (4-hydroxyphenylacetic and homovanillic acids). ^2^ Flavonoids comprise chalcones (xanthohumol), flavanols (catechin, epicatechin, procyanidin, and prodelphinidin dimers/trimers), flavanones (isoxanthohumol and naringin), flavones (apigenin), flavonols (myricetin and quercetin), and isoflavonoids (biochanin A). ^3^ Lignans comprise matairesinol and secoisolariciresinol.
